# Detection of Antibiotic Residues in Blossom Honeys from Different Regions in Turkey by LC-MS/MS Method

**DOI:** 10.3390/antibiotics11030357

**Published:** 2022-03-08

**Authors:** Buket Er Demirhan, Burak Demirhan

**Affiliations:** Department of Pharmaceutical Basic Sciences, Faculty of Pharmacy, Gazi University, Ankara 06330, Turkey; bdemirhan@gazi.edu.tr

**Keywords:** blossom honey, antibiotic, food safety, LC/MS-MS

## Abstract

In the present study, a total of 80 commercial blossom honey samples were obtained from local markets in Ankara, Turkey. These honeys were analyzed for 35 important and risky antibiotics (sulfonamide, tetracycline, macrolide, cephalosporin, aminoglycoside, quinolone, nitrofuran, chloramphenicol, and anthelmintic groups) by the LC-MS/MS multi-antibiotic method. In addition to these analyses, pH measure, moisture, and electrical conductivity were determined in these honey samples. Finally, seven out of 35 antibiotic residues investigated in the honeys were positive. The most frequently detected antibiotics in the analyzed samples were dihydrostreptomycin, streptomycin, erythromycin, sulfadimidine (sulfamethazine), and enrofloxacin as 58.75%, 22.5%, 13.75%, 10%, and 2.5%, respectively. Tetracycline and doxycycline were detected in only one sample. The pH, moisture, and electrical conductivity values of the honey samples were determined as between pH 3.78 and 5.41, 17.48 and 18.03%, and 0.25 and 0.47 mS/cm, respectively. In terms of food safety and human health, it is very important to monitor the residues of these pharmacologically active substances with analytical methods.

## 1. Introduction

Honey is defined as “a natural and sweet substance that occurs as a result of the nectar secreted from the flowers or other living parts of the plants and the secondary substances secreted by some insects living on the plant, after being collected by the honey bees (*Apis mellifera*) and modified by combining them with specific substances, reducing the water content and storing them in the honey-comb and maturing them” [1]. Honey, which is the oldest foodstuff, was used as the main sweetener in the world for a long time, until sugar cane was cultivated [2,3]. Honey has several health-beneficial effects, such as antibacterial, antifungal, and anti-inflammatory effects [4]. This natural product is synthesized from the nectar of flowers by honey bees [5,6,7]. The consumption of honey is widespread throughout the world, as it is healthy and nutritious, but in some cases, it may contain contaminants that can pose serious health risks [8]. Honey, which is a healthy food with high nutritional value, must be free from chemical pollutants such as antibiotics in terms of food safety [9]. In terms of public health concerns, food safety is a common problem worldwide. Therefore, there is a current trend to monitor and control veterinary medicines used in farm animals raised for food production [10].

Microorganisms and chemical pollutants are important sources of risk for human health [11]. With the increase of industrialization, pollution such as metals and antibiotics in environmental matrices such as water and soil are important environmental problems [12,13]. Especially, antibiotic residues have come to the forefront as the chemical pollutants in foods [11]. Antibiotics are natural, semisynthetic, or synthetic drugs and have antimicrobial activity [14]. These antibacterial drugs can be used for prophylactic or therapeutic purposes to treat disease in animals and as growth-promoting agents in subtherapeutic concentrations [14,15,16,17]. The use of antibiotics in animals can create residues in foods of animal origin, such as meat and honey [18,19]. Antibiotics can be found in honey, as they are used in beekeeping for the treatment of bacterial diseases. Generally, tetracyclines, streptomycin, sulfonamides, and chloramphenicol can be widely used in the control of bee brood diseases [8]. It is stated that, as a result of the use of antimicrobials, high levels of residues can occur in honey in a short period, usually within a week. Many of the antimicrobial substances cannot be metabolized by honey bees, and it is stated that drug residues can be found in honey harvested after drug application, even after a long time. The presence of antimicrobial residues in honey has many disadvantages. While some antibiotics have toxic effects, residues of some antibiotics can cause side effects in consumers, such as an allergic reaction or inhibition of skeletal growth [20]. In particular, the first concern about the release of antibiotics into the environment is associated with the risk of developing antimicrobial resistance among microorganisms [10]. Therefore, maximum residue limits (MRLs) for antibiotics have been established in animal origin foods produced from animals treated with various antibiotics, such as sulfonamides and tetracyclines. However, there are no established MRLs on antibiotic residues in honey [8]. In addition, there are no Maximum Residue Limits (MRLs) established for antibiotics in honey in the Turkish Food Codex (TFC).

Several quality parameters of honey such as color, electrical conductivity, pH, enzyme activity, ash contents, and even taste of honey vary with the honey bee species, geo-graphical origin, and presence of impurities [21]. The pH value also indicates the purity or crudeness of honey, but it depends upon the geographical area [3]. Conductivity is a frequently used criterion in routine honey quality control and is used to evaluate the botanical origin and purity of honey. The concentrations of mineral salts, organic acids, and proteins of honey affect the electrical conductivity of honey. The source of the flower, the amount of organic acid, the amount of protein, and the storage time are among the other factors that affect the electrical conductivity of honey [22]. One of the important parameters affecting the quality of honey is humidity. The water level of honey is effective in the stability of honey against fermentation and granulation. Therefore, honey with a high water content can ferment quickly [23].

The aim of the present study was to investigate the antibiotic presence and some quality properties in blossom honey samples obtained at Ankara local markets in Turkey.

## 2. Results

**Figure 1:**
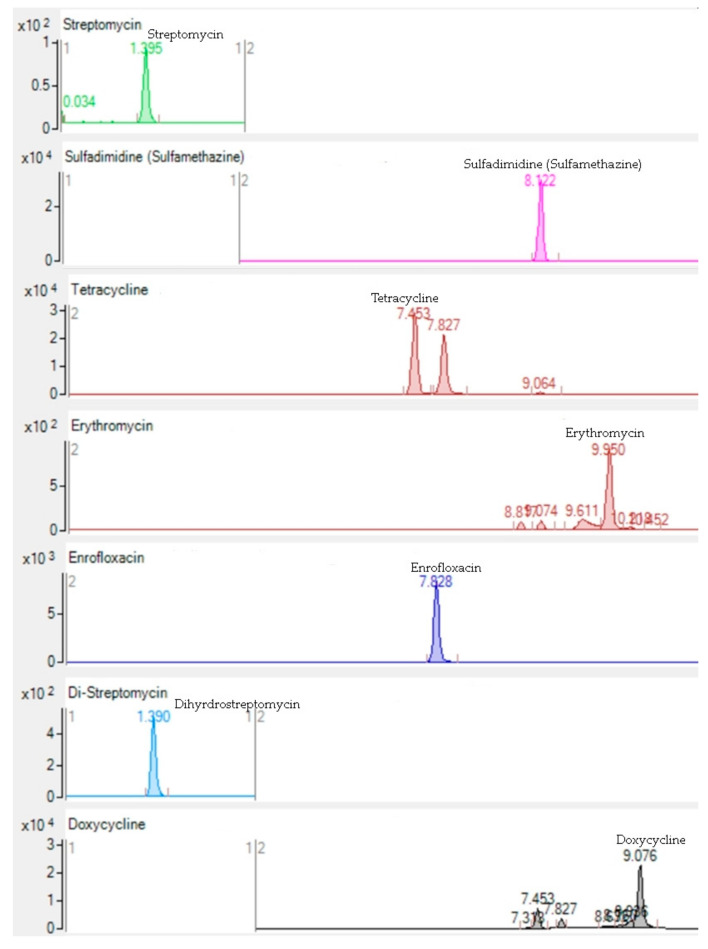
LC-MS/MS chromatograms (X-axis = retention time, min. and Y-axis = response) of antibiotics detected positive: streptomycin, sulfadimidine (sulfamethazine), tetracycline, erythromycin, enrofloxacin, dihydrostreptomycin, and doxycycline.

A total of 80 honey samples (eight different brands: A, B, C, D, E, F, G, and H) were analyzed for the thirty-five antibiotic residues (sulfonamide, tetracycline, macrolide, cephalosporin, aminoglycoside, quinolone, nitrofuran, chloramphenicol, and anthelmintic groups) by LC-MS/MS and pH, moisture, and electrical conductivity. Validation parameters of the applied LC-MS/MS multi-antibiotic method were used as performance criteria for method validation. Validation parameters such as relative standard deviation (RSD), recovery (Rec), the limit of detection (LOD), the limit of quantification (LOQ), and determination coefficient (R^2^) were assessed (Table 1). The LC-MS/MS chromatograms of the antibiotics are shown in Figure 1.

Positive antibiotic residue values of honey are given in Table 2. Enrofloxacin antibiotic residue was detected as 4.85 and 7.03 μg/kg in one sample in the G and H brands, respectively. Tetracycline and doxycycline antibiotic residues were also detected in the G brand as 1.36 μg/kg and 2.49 μg/kg, respectively, in only one sample. Among the brands, B brand had the highest dihydrostreptomycin antibiotic residue, and the lowest dihydrostreptomycin antibiotic residue was detected in H brand. The difference between the brands in terms of dihydrostreptomycin antibiotic residue was not statistically significant (*p* > 0.05). The difference between the brands in terms of streptomycin antibiotic residue was not statistically significant (*p* > 0.05). G brand had the highest streptomycin antibiotic residue, and B brand had the lowest streptomycin antibiotic residue in positive samples. Streptomycin antibiotic residues were not detected in the A and D brands. Streptomycin antibiotic residue of H brand was 6.18 μg/kg in one sample. The difference between the mean sulfadimidine (sulfamethazine) antibiotic residues of brands B and E was found to be statistically significant (*p* < 0.05). No antibiotic residues of sulfadimidine (sulfamethazine) were found in the A, C, F, G, and H brands. Sulfadimidine (sulfamethazine) residue was detected as 19.64 μg/kg in one sample in the D brand. Erythromycin residue was not detected in the A, B, C, D, and E brands. H brand has the highest erythromycin residue among the F, G, and H brands (*p* < 0.05). The difference between the mean values of erythromycin antibiotic residues of the F and G brands was not statistically significant (*p* > 0.05).

The pH, moisture, and electrical conductivity values of the brands are given in Table 3. The water content of honey is mainly due to nectar. Honey harvest time, the percentage of glazed comb, climatic conditions during harvest and storage conditions also affect the water content of honey. The water content affects the quality, viscosity, crystallization, and taste of honey and is accepted as a criterion in determining the shelf life and maturity level of honey [24]. According to the Turkish Food Codex (TFC) regulation on honey, the moisture content of blossom honey should not be more than 20% [25]. The moisture values of the honey were found within the limit values specified in the TFC. The difference between brands in terms of moisture was not statistically significant (*p* > 0.05). The B brand had the highest moisture content, followed by the G, F, D, H, E, and C brands. The A brand had the lowest moisture content. 

According to the Turkish Standards Institute (TSE), the pH value of honey should be between 3.4 and 6.1 [1]. The pH values of the analyzed honey were found within the limits specified by the TSE. The difference between the brands in terms of pH values was found to be statistically significant (*p* < 0.05). Brand B had the highest pH value, followed by brands D, H, F, A, G, and C. The E brand had the lowest pH value.

Electrical conductivity is used to distinguish between secretory honey and blossom honey. The electrical conductivity of honey can also vary according to the mineral and acid contents they contain. The electrical conductivity of secretory honey may also be higher than blossom honey [24]. According to the honey standard in the TSE, the electrical conductivity of blossom honey should be lower than 0.8 mS/cm [1]. The electrical conductivity of the analyzed honey was found to be in accordance with the values specified in the TSE. The difference between the brands in terms of electrical conductivity values was found to be statistically significant (*p* < 0.05). The B brand had the highest electrical conductivity value, followed by the H, A, D, E, G, and C brands. It was determined that the F brand had the lowest electrical conductivity value.

## 3. Discussion

It is stated that antibiotics used in the treatment of various diseases seen in honey bees cause residue problems in honey [26]. Unfortunately, only the maximum residue limit for amitraz and coumaphos is specified in honey, and these values are indicated as 200 µg/kg and 100 µg/kg, respectively, in the Turkish Food Codex Regulation on Classification and Maximum Residue Limits of Pharmacologically Active Substances in Food-stuffs of Animal Origin [27]. Studies on antibiotic residues in honey have been evaluated in the international literature. Yang et al. [28] developed the UPLC-MS/MS method for the detection of 70 antibiotics in honey and analyzed fifty honey samples to evaluate the applicability of the method. It was stated that they detected metacycline, oxytetracycline, and tetracycline residues and its metabolite 4-tetracycline residue in these samples. They detected tetracycline antibiotic in the concentration range of 2.6 μg/kg–215.3 μg/kg in three samples and 4-tetracycline metabolite at concentrations of 4.6 μg/kg and 232.7 μg/kg in two samples. They reported that they detected oxytetracycline and metacycline antibiotics at a concentration of 4.0 µg/kg and 159.9 µg/kg, respectively, in one sample. They report that these results may be related to easy access, cheapness, the flexibility of use, or increasing the normal dose to improve the efficacy of antibiotics. In our study, the tetracycline antibiotic residue was 1.36 μg/kg in a sample of the G brand, and the tetracycline residue was found to be low according to the results of Yang et al. [28].

Bonerba et al. [29] investigated antibiotic residues in honey by liquid chromatography High-Resolution Mass Spectrometry. They reported that the antibiotic residue concentrations detected in honey samples were determined as 18.6 ng/g in 33 positive samples of streptomycin antibiotic residue by the Randox II screening test. They stated that, with the Randox IV screening test, erythromycin antibiotic residue was found to be 4.4 ng/g in 35 positive samples and 13.1 ng/g for streptomycin residue in 65 positive samples. Savarino et al. [30] stated in their study that they found erythromycin antibiotic residue in six samples to be 7.3 ± 0.73 ppb and streptomycin antibiotic residue in 20 samples from experimental apiary honey as 23.3 ± 8.6 ppb in Altamura honey. In our study, erythromycin antibiotic residue was found to be 59.62 ± 18.73 μg/kg, 78.32 ± 36.12 μg/kg, and 358.11 ± 6.37 μg/kg in three brands (F, G, and H), respectively. It was observed that the streptomycin antibiotic residue ranged between n.d. and 6.20 ± 2.96 μg/kg in the brands. When compared with the values of Bonerba et al. [29] and Savarino et al. [30], erythromycin antibiotic residue was found to be high, and streptomycin antibiotic residue was found to be low in our study. Kumar et al. [19] reported that eight (5.3%) out of 150 honey samples in India were positive for erythromycin residues, and they found the mean ± SD to be 78.8 ± 23.6 ng/g. They reported that erythromycin antibiotic residue in positive samples was in the range of 50–112 ng/g. Similar to Kumar et al. [19], in our study, the mean erythromycin antibiotic residue of the G and F brands was the same, and the H brand had high erythromycin antibiotic residue. They stated that the pharmacologically active substances in their studies are widely used in veterinary medicine, and therefore, environmental contamination (spread with urine and feces) becomes possible. They also stated that pharmacologically active substances in the soil can reach different parts of the plant, or antibiotic residues may occur as a result of the addition of antibiotics such as sulfonamides and tetracyclines to the drinking water of farm animals and the consumption of these waters by honey bees. Veterinary antibiotic preparations are sold by prescription in our country. Since the unconscious use of antibiotics will be limited, it is thought that excessive use of antibiotics for the diseases of honey bees may cause this situation.

Mahmoudi et al. [31] investigated antibiotic residues in 135 honey samples collected in different seasons. They stated that the most frequently detected antibiotic residue was enrofloxacin, and they found it in the range of 0.6–72.1 ng/g. In our study, enrofloxacin antibiotic residue was determined as 4.85 and 7.03 μg/kg in the G and H brands in one sample, and it was seen that the enrofloxacin antibiotic residue in the honey in our study was low compared to the values of the researchers. Tamba-Berehoiu et al. [32] analyzed streptomycin, tetracycline, and erythromycin residues in acacia honey, linden honey, and polyfloral honey. They found the streptomycin residues in acacia, linden, and polyfloral honey as 50.88 ppb, 42.77 ppb, and 51.49 ppb, respectively. They found the erythromycin antibiotic residues in Acacia, linden, and polyfloral honey as 0.27 ppb, 0.11 ppb, and 0.06 ppb, respectively. When compared with the values of Tamba-Berehoiu et al. [32], erythromycin antibiotic residue was found to be high, and streptomycin antibiotic residue was found to be low in our study. Vidal et al. [33] stated that they detected 8.6 µg/kg erythromycin antibiotic residue in only one of the commercial honey samples. They also reported that the antibiotic residue of sulfadimidine was lower than the LOQ value in a sample they obtained from the beekeeper. It is stated that the detection of multiple antibiotic residues in the same sample is important for the analysis of drug residues in honey. Researchers have also reported that drug residues in honey may occur as a result of the wrong beekeeping practices, considering the zero tolerance policy. They have stated that more work needs to be done for this purpose. Reybroeck [34] stated that he found streptomycin residues in four (1.6%) out of 248 samples and sulfonamide residues in three (4.2%) out of 72 samples in his study in which antibiotic residues were investigated in local honey samples. He reported that he found streptomycin residues in 51 (47.2%) out of 108 samples and sulfonamide antibiotic residues in 31 (31.6%) out of 98 samples in imported honey samples. Reybroeck [34] found that, while the streptomycin residue in local honeys was lower than the value in our study, the percentage of streptomycin in imported honeys was higher than the percentage of streptomycin in our study.

There have also been studies on antibiotic residues in honey in Turkey. Ağaoğlu et al. [35] analyzed the residues of tetracycline and streptomycin group antibiotics in packaged and open honey. They found the mean streptomycin residues to be 25.8 ± 10.8 μg/kg and 8.21 ± 5.2 μg/kg in packaged and open honey, respectively. This streptomycin residue level appears to be higher than the streptomycin residue in our study. They also reported that, although illegal, some drugs can be used in beekeeping, or bees can be exposed to antibiotics added to the feed or water of other animals. It is stated by researchers that the residual level and positivity rate of honey poses a potential risk to the consumer, and in this context, it may be beneficial to take measures such as raising awareness of beekeepers about production, controlling drug sales to beekeepers, making more frequent controls by the competent authorities, and imposing necessary sanctions when antibiotics are detected above the maximum residue limits. Çakar and Gürel [36] stated that they did not find any antibiotic residues in honey in their study, where they investigated 20 sulfonamides and five tetracycline group antibiotic residues in honey. Kutlu et al. [37] analyzed six tetracyclines and 10 sulfonamide group antibiotic residues in honey and reported that they did not detect antibiotic residues in honey samples. Özkan et al. [38] investigated the antibiotic residues in honey and stated that they found streptomycin residues in 37% and sulfonamide residues in 52% of the samples. This 37% residual streptomycin level appears to be slightly higher than the streptomycin percentage in our study. Researchers have emphasized that informing honey beekeepers about residue problems is important for both economic and consumer health and the importance of preventing risky products from being offered for consumption with routine controls.

According to the beekeeping regulation in our country, beekeepers must comply with the relevant legislation in the use of veterinary medicinal products, record the veterinary medicinal products they use, keep the prescriptions, and submit them to the Ministry during inspections [39].

## 4. Materials and Methods

### 4.1. Samples Collection

A total of 80 commercial blossom honey samples of different brands (A, B, C, D, E, F, G, and H) before their expiry date and with the date of collected from April to July 2021 that is obtained from several local markets in Ankara, Turkey. Brands of collected blossom honey samples are also sold all over Turkey. According to the labels of the blossom honey samples analyzed for antibiotic residues, the sources of honey are from different regions of Turkey, including the Aegean, Mediterranean, Central Anatolia, Southeastern Anatolia, and Eastern Anatolia regions.

### 4.2. Sample Preparation

Analyzed sample preparations were done according to the instructions of the Jasem LC-MS/MS multi-antibiotic analysis kit (SEM, Istanbul, Turkey) [40]. For the analysis of multiple antibiotics in honey, 5.0 g of blossom honey sample was weighed and transferred to 50-mL centrifuge tubes. Two hundred microliters of international standard solution (ISTD) and 20 mL of reagent 1 (Jasem, JSM FO 2503, SEM, İstanbul, Turkey) were added to the samples and shaken with a multi-shaker for 15 min. After shaking, the tubes were centrifuged at 3000 rpm for 10 min at room temperature. Following centrifugation, the clear supernatant in the tube was removed, filtered through a 0.45-micron nylon filter into an HPLC vial, and injected directly into the LC-MS/MS device.

### 4.3. LC–MS/MS Analysis

Multiple antibiotics (sulfonamide, tetracycline, macrolide, cephalosporin, aminoglycoside, quinolone, nitrofuran, chloramphenicol, and anthelmintic groups) were screened in blossom honey samples with a commercial multi-antibiotic LC-MS/MS Jasem kit (SEM, Istanbul, Turkey) containing mobile phases, sample preparation reagent, standards, and the analytical column [40]. An antibiotic analysis was performed according to the manufacturer’s instructions. Multi-antibiotics were analyzed by using an Agilent LC 1290 combined with an Ultivo triple–quad mass spectrometer (LC-MS/MS) and Electrospray ionization (ESI) (Agilent Technologies, Santa Clara, CA, USA), equipped with a Jasem analytical column (JSM FO 2575, SEM, Istanbul, Turkey). The column furnace temperature was set to 35 °C. The flow rate was 0.4 mL/min, and the injection volume was 10 µL. AJS ESI ion source parameters used in the mass spectrometer were determined as the gas temperature 300 °C, nebulizer gas (N_2_) flow 6 L/min, nebulizer pressure 35 (psi), sheath gas temperature 400 °C, sheath gas (N_2_) flow 11 L/min, and capillary voltage positive +4500 V and negative −3500 V. It was studied with an antibiotic-free honey sample to create matrix effect calibrations. All analytes were weighed separately, and the main stocks were formed with methanol at 1000 mg/L. Then, 100-mg/L, 10-mg/L, and 1-mg/L intermediate stocks from all the main stocks and 10/20/50/100/200/500/1000-µg/L intermediate stocks were created from 1-mg/L intermediate stocks, respectively. Seven levels (1, 2, 5, 10, 20, 50, and 100 µg/L) of calibration solutions were prepared by taking 100 µL of each of the last intermediates and adding 20 µL of internal standard (10 ppm) and 880 µL of the blank matrix.

For data acquisition, method creation, and qualitative and quantitative analysis, a MassHunter Ultivo LC/TQ, version 1.2 (Agilent Technologies, Santa Clara, CA, USA), was used. The antibiotic levels in each honey sample were expressed as μg/kg. Parent ion and fragment ions, retention time, concentration ranges, fragmentor, and collision energies of the mycotoxins are presented in Table 4.

### 4.4. Determination of pH, Moisture, and Electric Conductivity

The pH, moisture, and electric conductivity of honey was determined according to the method described by the Turkish Standard Institute [1,41,42]. Ten grams of honey were weighed in a 100-mL beaker, and 75 mL of distilled water was added to the sample. Then, using a pH meter (Hanna 211) calibrated with appropriate buffers (7.0 pH solution and 4.0 pH solution) for each blossom honey sample, a direct reading was taken from the pH meter [1]. The moisture in honey was determined using the refractometric method according to the TSI. During the analysis, care was taken to ensure that the honey was at a temperature of approximately 20 °C [41]. For the electric conductivity, 20 g of honey was weighed in a 100-mL volumetric flask with distilled water. Take the reading in 40-mL samples as soon as the conductivity stabilizes. The electrical conductivity electrode was rinsed with distilled water at each sample change [42].

### 4.5. Statistics

The homogeneity and normality of the brands were determined by SPSS 16 (SPSS Inc., Chicago, IL, USA). Statistical evaluation was made with One-Way ANOVA using the Duncan method for brands with homogeneous distribution and with the Tamhane method for brands with nonhomogeneous distributions. The normality of the distribution of the sulfadimidine (sulfamethazine) data was determined by the Shapiro–Wilk test, and the Mann–Whitney *U* test was used for the statistical comparison of brands [43].

## 5. Conclusions

Traces of pharmacologically active substances must be detected in honey to gain knowledge of the risks threatening both humans and ecosystems and to identify the options needed to reduce these risks. The monitoring of antibiotic residues in honey is important in terms of evaluating the possible risk of these products to human health. Having a general idea of the antibiotics used during beekeeping enables the assessment of antibiotic residues and potential environmental pollution. The present study provides an overview of the maximum residue limits for antibiotics in honey, which is the major bee product, as well as the identification and determination of different antibiotics that may be potentially present in honey. There is no information about the maximum residual limits of antibiotic residues in honey, in the legal regulations in our country, as in many other countries. For this reason, we think that it is important to make a legal regulation that provides reliability and certainty about antibiotic residues in honey. At the same time, it is important to monitor the pollution concentrations in honey.

## Figures and Tables

**Table 1:** Validation parameters of the applied LC-MS/MS method.

Antibiotics	% RSD	% Rec	LOD (µg/kg)	LOQ (µg/kg)	R^2^
Albendazole	0.23	81.52	0.68	2.27	0.998
Chloramphenicol	0.90	55.11	2.69	8.97	0.998
Chlortetracycline	0.44	88.63	1.31	4.37	0.998
Ciprofloxacin	0.17	83.89	0.51	1.72	0.998
Dihydrostreptomycin	0.12	81.26	0.37	1.22	0.999
Doxycycline	0.48	77.36	1.45	4.83	0.993
Enrofloxacin	0.48	92.25	1.44	4.78	0.996
Erythromycin	0.56	79.45	1.68	5.61	0.992
Fenbendazole	0.23	95.12	0.70	2.32	0.994
Flubendazole	0.17	90.42	0.52	1.72	0.998
Furazolidon	0.16	80.45	0.47	1.56	0.999
Levamisole	0.09	88.49	0.27	0.89	0.998
Marbofloxacin	0.08	90.03	0.25	0.85	0.995
Mebendazole	0.18	90.27	0.54	1.80	0.995
Oxolinic acid	0.30	77.02	0.89	2.96	0.997
Oxytetracycline	0.31	82.08	0.93	3.10	0.996
Streptomycin	0.32	88.93	0.95	3.15	0.999
Sulfachloropyridazine	0.48	98.98	1.44	4.81	0.997
Sulfadiazine (Silvadene)	0.51	111.60	1.53	5.10	0.994
Sulfadimethoxine	0.42	107.86	1.27	4.24	0.998
Sulfadimidine (Sulfamethazine)	0.36	120.91	1.07	3.57	0.999
Sulfadoxine	0.30	98.86	0.89	2.95	0.998
Sulfamerazine	0.17	62.00	0.52	1.72	0.998
Sulfamethoxazole	0.44	89.50	1.33	4.42	0.996
Sulfapyridine	0.44	106.28	1.32	4.41	0.999
Sulfaquinoxaline	0.45	108.88	1.35	4.50	0.994
Sulfathiazole	0.49	111.93	1.47	4.91	0.993
Tetracycline	0.28	89.97	0.83	2.78	0.999
Thiabendazole	0.12	84.63	0.36	1.21	0.998
Tylosin	0.23	80.10	0.70	2.33	0.999
5-Hydroxy-Thiabendazole	0.23	99.97	0.70	2.34	0.999
Cefquinome	0.53	84.20	1.59	5.32	0.992
Ceftiofur	0.46	78.52	1.39	4.65	0.998
Cephapirin	0.21	80.00	0.64	2.12	0.997
Emamectin B1a	0.44	73.00	1.31	4.37	0.999

**Table 2:** Antibiotic residues of the brands (mean ± SE).

Brands	Dihydrostreptomycin (μg/kg)	Streptomycin (μg/kg)	Sulfadimidine (Sulfamethazine) (μg/kg)	Erythromycin (μg/kg)
A	8.22 ± 4.67	nd	nd	nd
B	76.66 ± 32.59	1.22 ± 0.02	6.62 ± 1.41 ^a^	nd
C	50.75 ± 20.28	4.02 ± 1.54	nd	nd
D	8.82 ± 3.43	nd	19.64	nd
E	6.89 ± 1.35	2.35 ± 1.09	1.36 ± 0.12 ^b^	nd
F	31.11 ± 8.47	2.15 ± 0.74	nd	59.62 ± 18.73 ^b^
G	22.15 ±13.92	6.20 ± 2.96	nd	78.32 ± 36.12 ^b^
H	4.40 ± 0.95	6.18	nd	358.11 ± 6.37 ^a^

Mean values with different letters in the same column are statistically different (*p* < 0.05).

**Table 3:** The pH, moisture, and electrical conductivity of the honey samples (mean ± SE).

Brands	pH	Moisture (%)	Electrical Conductivity (mS/cm)
A	3.98 ± 0.02 ^c^	17.48 ± 0.25	0.38 ± 0.02 ^bcd^
B	5.41 ± 0.05 ^a^	18.03 ± 0.08	0.47 ± 0.01 ^a^
C	3.82 ± 0.01 ^d^	17.70 ± 0.08	0.32 ± 0.002 ^d^
D	4.21 ± 0.03 ^b^	17.85 ± 0.13	0.37 ± 0.01 ^bc^
E	3.78 ± 0.02 ^d^	17.78 ± 0.08	0.35 ± 0.02 ^cd^
F	3.99 ± 0.04 ^c^	17.88 ± 0.13	0.25 ± 0.01 ^e^
G	3.85 ± 0.01 ^d^	17.90 ± 0.16	0.32 ± 0.01 ^d^
H	4.04 ± 0.04 ^c^	17.78 ± 0.22	0.46 ± 0.03 ^ab^

Mean values with different letters in the same column are statistically different (*p* < 0.05).

**Table 4:** Optimized MS/MS parameters for the mycotoxins.

Antibiotics	RT	Parent ion (*m/z*)	Fragment Ions			Concentration Range (µg/L)	Ion Mode	Fragmentor Voltage (V)	CE (V)		
		Q1	Q2	Q3	Q4						
Albendazole	10.58 ± 0.01	266.1	234	191	-	1–100	Positive	155	16	32	-
Chloramphenicol	9.85 ± 0.01	321	257	152	-	1–100	Negative	113	0	4	-
Chlortetracycline	8.37 ± 0.03	479.1	462	260	444	1–100	Positive	120	6	68	22
Ciprofloxacin	7.52 ± 0.02	332	314	231	-	1–100	Positive	130	20	35	-
Dihydrostreptomycin	1.39 ± 0.05	584.5	263	246	-	1–100	Positive	190	32	42	-
Streptomycin	1.39 ± 0.05	582.2	263	246	221	1–100	Positive	170	36	44	46
Doxycycline	9.07 ± 0.01	445.2	428	154	-	1–100	Positive	110	2	18	-
Enrofloxacin	7.82 ± 0.01	360	342	316	342	1–100	Positive	120	20	16	20
Erythromycin	9.95 ± 0.01	734.5	576	158	-	1–100	Positive	150	16	32	-
Fenbendazole	10.52 ± 0.02	300.1	268	159	-	1–100	Positive	156	20	36	-
Flubendazole	10.93 ± 0.01	314.1	282	123	-	1–100	Positive	146	20	36	-
Furazolidon	8.81 ± 0.01	226	122	67	-	1–100	Positive	150	20	40	-
Levamisole	6.83 ± 0.04	205.1	178	91.1	-	1–100	Positive	141	20	44	-
Marbofloxacin	7.29 ± 0.01	363	345	320	-	1–100	Positive	120	17	9	-
Mebendazole	10.63 ± 0.11	296.1	264	77	-	1–100	Positive	151	20	48	-
Oxolinic acid	9.90 ± 0.01	262.1	244	216	-	1–100	Positive	114	12	28	-
Oxytetracycline	7.39 ± 0.01	461.2	443	426	-	1–100	Positive	90	10	16	-
Sulfachloropyridazine	9.05 ± 0.09	285	156	92.1	-	1–100	Positive	108	12	24	-
Sulfadiazine (Silvadene)	6.83 ± 0.01	251.1	156	92.1	-	1–100	Positive	96	8	28	-
Sulfadimethoxine	10.32 ± 0.01	311.1	156	108	-	1–100	Positive	128	16	28	-
Sulfadimidine (Sulfamethazine)	8.12 ± 0.01	279.1	186	124	156	1–100	Positive	80	16	32	18
Sulfadoxine	9.32 ± 0.09	311.1	156	92.1	-	1–100	Positive	126	16	32	-
Sulfamerazine	7.58 ± 0.02	265.1	156	-	-	1–100	Positive	114	12	-	-
Sulfamethoxazole	9.43 ± 0.01	254.1	156	92.1	-	1–100	Positive	108	12	24	-
Sulfapyridine	7.31 ± 0.01	250.1	108	92	-	1–100	Positive	150	20	20	-
Sulfaquinoxaline	10.32 ± 0.04	301.1	156	92	-	1–100	Positive	118	16	32	-
Sulfathiazole	7.18 ± 0.03	256	156	92.1	-	1–100	Positive	102	12	28	-
Tetracycline	7.45 ± 0.01	445.2	154	427	410	1–100	Positive	100	28	8	16
Thiabendazole	7.03 ± 0.05	202	175	131	-	1–100	Positive	130	24	36	-
Tylosin	10.19 ± 0.09	916.5	101	174	772	1–100	Positive	230	56	44	32
5-Hydroxy-Thiabendazole	6.53 ± 0.10	218	147	234	-	1–100	Positive	120	40	40	-
Cefquinome	6.89 ± 0.11	529.1	396	134	-	1–100	Positive	110	8	12	-
Ceftiofur	9.47 ± 0.12	524	241	127	126	1–100	Positive	141	16	48	40
Emamectin B1a	12.10 ± 0.13	886.5	158	82.1	-	1–100	Positive	190	40	60	-

RT: Retention time and CE: Collision energies.

## Data Availability

The datasets analyzed during the study are included in the article.
